# External Validation of the Machine Learning-Based Thermographic Indices for Rheumatoid Arthritis: A Prospective Longitudinal Study

**DOI:** 10.3390/diagnostics14131394

**Published:** 2024-06-30

**Authors:** Isabel Morales-Ivorra, Delia Taverner, Oriol Codina, Sonia Castell, Peter Fischer, Derek Onken, Píndaro Martínez-Osuna, Chakib Battioui, Manuel Alejandro Marín-López

**Affiliations:** 1 Rheumatology Department, Hospital Universitari d’Igualada, 08700 Igualada, Spain; 2 Rheumatology Department, Hospital Universitari Sant Joan de Reus, 43204 Reus, Spain; 3 Rheumatology Department, Hospital de Figueres, 17600 Figueres, Spain; 4 Immunology, Eli Lilly and Company, Indianapolis, IN 46225, USA; 5 Advanced Analytics and Data Sciences, Eli Lilly and Company, Indianapolis, IN 46225, USA; 6 R+D Department, Singularity Biomed, 08174 Sant Cugat del Vallès, Spain; manuel.marin@singularity-biomed.com

**Keywords:** rheumatoid arthritis, thermography, external validation, machine learning, artificial intelligence

## Abstract

External validation is crucial in developing reliable machine learning models. This study aimed to validate three novel indices—Thermographic Joint Inflammation Score (ThermoJIS), Thermographic Disease Activity Index (ThermoDAI), and Thermographic Disease Activity Index-C-reactive protein (ThermoDAI-CRP)—based on hand thermography and machine learning to assess joint inflammation and disease activity in rheumatoid arthritis (RA) patients. A 12-week prospective observational study was conducted with 77 RA patients recruited from rheumatology departments of three hospitals. During routine care visits, indices were obtained at baseline and week 12 visits using a pre-trained machine learning model. The performance of these indices was assessed cross-sectionally and longitudinally using correlation coefficients, the area under the receiver operating curve (AUROC), sensitivity, specificity, and positive and negative predictive values. ThermoDAI and ThermoDAI-CRP correlated with CDAI, SDAI, and DAS28-CRP cross-sectionally (ρ = 0.81; ρ = 0.83; ρ = 0.78) and longitudinally (ρ = 0.55; ρ = 0.61; ρ = 0.60), all *p* < 0.001. ThermoDAI and ThermoDAI-CRP also outperformed Patient Global Assessment (PGA) and PGA + C-reactive protein (CRP) in detecting changes in 28-swollen joint counts (SJC28). ThermoJIS had an AUROC of 0.67 (95% CI, 0.58 to 0.76) for detecting patients with swollen joints and effectively identified patients transitioning from SJC28 > 1 at baseline visit to SJC28 ≤ 1 at week 12 visit. These results support the effectiveness of ThermoJIS in assessing joint inflammation, as well as ThermoDAI and ThermoDAI-CRP in evaluating disease activity in RA patients.

## 1. Introduction

Rheumatoid arthritis (RA) is a chronic inflammatory disease that causes persistent joint inflammation. RA is characterised by significant pain, loss of joint function, disability, and reduced quality of life. Current therapies, combined with the implementation of treat-to-target and tight control strategies, have been shown to lead to improved outcomes and make remission an achievable goal [1,2,3,4,5]. The cardinal signs of inflammation include pain, swelling, heat, and redness. Pain and swelling are typically the primary focus of the physical examination when assessing inflammation. However, recent studies indicate that the warmth caused by synovial inflammation can also be a useful marker of joint inflammation [6,7,8,9,10,11,12,13,14,15,16].

Despite advances in RA assessment, accurate and timely detection of joint inflammation remains a challenge. While current imaging techniques, such as ultrasound and Magnetic Resonance Imaging (MRI), are effective, they can be time-consuming, costly, and require specialised training. Consequently, these methods are often not feasible for routine clinical practice. Therefore, there is a need for a rapid, affordable, and accurate method to assess joint inflammation in patients with RA.

Thermography is a fast, non-invasive imaging technique that uses thermal cameras to capture the intensity of long-wave infrared radiation emitted by bodies, generating pictures of the heat pattern [17,18,19]. Therefore, thermography represents a potentially valuable tool for the detection of arthritis [20,21,22]. In our previous work, we developed and trained a machine learning-based computational method that analyses thermal images of hands to assess joint inflammation. The resulting machine learning model produces a score named the Thermographic Joint Inflammation Score (ThermoJIS) [23]. In addition, we expanded the functionality of ThermoJIS by incorporating the Patient Global Assessment of the disease and acute phase reactants to develop two novel disease activity indices, namely Thermographic Disease Activity Index (ThermoDAI) and Thermographic Disease Activity Index C-reactive protein (ThermoDAI-CRP) [24]. Our initial validation with a hold-out dataset (independent from the training and tuning set) yielded promising results, but further external validation across diverse populations and clinical settings is crucial to confirm these results. Moreover, our previous studies were cross-sectional, so longitudinal studies are needed to evaluate sensitivity to change.

The aims of this study were to assess the effectiveness of ThermoJIS, ThermoDAI, and ThermoDAI-CRP on a new, independent prospective dataset and to evaluate their sensitivity to change.

## 2. Materials and Methods

### 2.1. Recruited Patients

The study design was a non-interventional prospective observational study conducted for 12 weeks. The study population comprised 77 RA consecutive patients recruited at outpatient visits to the departments of rheumatology of three hospitals between April and November 2022. Follow-up visits were conducted at week 12. The inclusion criteria were age over 18 years and a diagnosis of RA, as defined by the 2010 ACR/European League Against Rheumatism (EULAR) classification criteria [25]. Exclusion criteria were subjects with wounds, infections, or trauma on the dorsal side of the hands and those using bandages, cosmetics, or other substances that could affect the thermal pattern prior to data collection.

Diagnosis and management of the RA were performed according to the standard of care. The study was conducted in accordance with the ethical principles of the Declaration of Helsinki and Good Clinical Practice guidelines. Approval for human participant involvement was obtained from the following ethics committees: (1) CEIm del Hospital Universitari de Bellvitge (ID: ICPS009/22), (2) Comissió de Recerca del CSA (ID: PR2/2022), (3) CEIM GIRONA (ID: SB-TIMAG-21-02/2022.125), and (4) CEIm del Instituto d’Investigació Sanitària Pere Virigili (ID: 138/2022). Participants gave written informed consent to participate in the study before taking part.

### 2.2. Thermography Tests

All patients underwent thermography of both hands using a Thermal Expert TE-Q1 camera (i3system Inc., Daejeon, Korea) with a 6.8 mm lens. Thermal cameras were connected to a smartphone, and a custom mobile application was developed to acquire the raw thermal images, which corresponded to the intensity of infrared waves (see an image of the mobile application in Figure S1). To replicate real-world conditions, thermography was conducted during outpatient visits without an acclimatisation process or controlled room temperature. Thermal imaging was performed before the physical examination. The dorsal images of both hands were recorded with the fingers spread. No fixed distance between the camera and the hand was required. The researcher was instructed to frame and focus the image adequately to include all the joints in each hand. Subsequently, all the thermal images were analysed to obtain the ThermoJIS values. The results of ThermoJIS were not available to the researchers before the clinical examination.

### 2.3. Clinical and Laboratory Assessments

Clinical assessments were performed and included the number of swollen and tender joints in the standard 28-joint count examination (SJC28 and TJC28, respectively), as well as the Patient Global Assessment (PGA), the Evaluator Global Assessment (EGA) of disease activity based on a visual analogue scale score (VAS 0–10), the Health Assessment Questionnaire for Rheumatoid Arthritis (HAQ-DI) [26,27], and treatment-related data were collected at both the baseline and week 12 follow-up visits. Additionally, laboratory analyses were conducted, including the erythrocyte sedimentation rate (ESR) and C-reactive protein value (CRP). Response to treatment was evaluated with ACR20 and ACR50 response (defined as a 20% and 50%, respectively, reduction in the number of tender and swollen joints, as well as improvement in at least three of the other five ACR components) [28] and EULAR response criteria [29].

### 2.4. Calculation of ThermoJIS, ThermoDAI and ThermoDAI-CRP

ThermoJIS values were calculated using Singularity Biomed’s in-house software (v1.1, Singularity Biomed, Sant Cugat del Vallès, Spain) as follows: Initially, the images were processed for contrast enhancement, background removal, and noise reduction. Subsequently, a collection of local thermal features comprising unique inflammation patterns was extracted. Finally, a fixed deterministic machine learning model was used to analyse the features and determine the ThermoJIS value. Appendix A explains the previous development of the machine learning model. A higher ThermoJIS value corresponded to a greater probability of active synovitis.

ThermoDAI and ThermoDAI-CRP were defined as the simple linear sum of the outcome parameters: ThermoJIS, PGA, and for ThermoDAI-CRP, the CRP in mg/dL. Thus, the formulas for ThermoDAI and ThermoDAI were defined as follows:

ThermoDAI = ThermoJIS + PGA(1)

ThermoDAI-CRP = ThermoJIS + PGA + CRP(2)

ThermoJIS values and CRP (mg/dL) are limited from 0 to 10. Therefore, ThermoDAI and ThemoDAI-CRP range from 0 to 20 and from 0 to 30, respectively.

### 2.5. Statistical Analysis

Subject characteristics were described using means with standard deviations (SD), medians with interquartile ranges (IQR), or frequencies with proportions, as appropriate. The Shapiro–Wilk test was used to assess the normality of the data distributions. To calculate the statistical significance of differences between baseline and week 12, the paired t-test or Wilcoxon signed rank was used. McNemar’s test was also used to detect changes in proportions. The number of participants exceeded the minimum sample size calculations to provide robust results. Increased data in external validations enhances the generalisability and reliability of the findings. Correlations between scores were calculated using either Spearman’s rank correlation coefficient or Pearson’s correlation coefficient. For binary classifications, the area under the receiver operating characteristic curve (AUROC), contingency table, and related metrics such as positive predictive value (PPV), negative predictive value (NPV), sensitivity, and specificity were used. Cross-sectional analyses were conducted using the aggregate of visits from both baseline visits and week 12 visits. Patient visits with missing data were excluded. The Mann–Whitney U test was used to assess statistical significance in the non-normal distribution of independent samples. Statistical significance was determined with *p*-values below 0.05 (two-sided). The statistical analysis was performed using Python (v3.11).

## 3. Results

### 3.1. Patient Characteristics

Four patients missed the week 12 visit, resulting in a total of 73 patients completing the study. All subjects tolerated the procedure well, and no adverse effects were observed. Table 1 presents the demographic, clinical, laboratory, disease activity, and thermographic characteristics of the patients at both the baseline visit and the week 12 visit. Distributions of ThermoJIS, ThermoDAI, and ThermoDAI-CRP are shown in Figure S2.

### 3.2. Association of ThermoJIS with Swollen Joint Count

**Figure 1:**
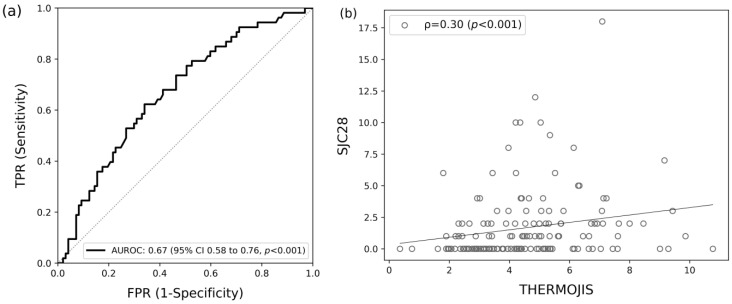
Association between the Thermographic Joint Inflammation Score (ThermoJIS) and the 28-swollen joint counts (SJC28). (**a**) Area under the receiver operating curve (AUROC) of the ThermoJIS for detecting swollen joints considered as SJC28 > 1 (AUROC, 0.67; 95% CI 0.58–0.76, *p* < 0.001). (**b**) Spearman’s correlation between ThermoJIS and SJC28 (ρ = 0.30, *p* < 0.001). Aggregated data from baseline visit and week 12 visit (n = 150).

ThermoJIS was evaluated for its ability to detect swollen joints defined as SJC28 > 1, using the aggregate of both baseline and week 12 visits and had an AUROC of 0.67 (95% CI, 0.58 to 0.76; *p* < 0.001) (Figure 1a). The Spearman’s correlation between ThermoJIS and SJC28 was 0.30 (*p* < 0.001) (Figure 1b). Using a cutoff threshold of 3.56, decided a priori as the optimal threshold with good sensitivity [23], ThermoJIS showed a sensitivity of 81%, specificity of 41%, positive predictive value (PPV) of 43%, and negative predictive value (NPV) of 80%.

### 3.3. Sensitive Detection of Synovitis

**Figure 2:**
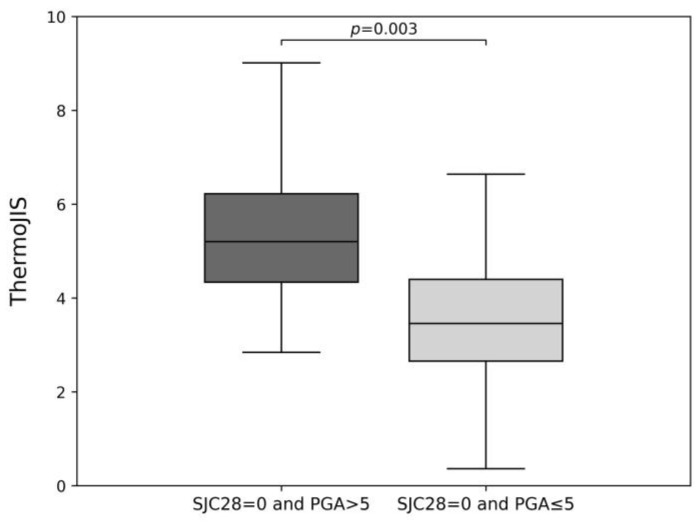
Indirect analysis of the Thermographic Joint Inflammation Score (ThermoJIS) performance in detecting synovitis that is not evident through swollen joint count by comparing the ThermoJIS values of patients with 28-swollen joint counts (SJC28) = 0 and Patient Global Assessment (PGA) >5 (n = 11) versus patients with SJC28 = 0 and PGA ≤ 5 (n = 62). The Mann–Whitney U test was used to assess statistical significance.

In order to indirectly evaluate whether ThermoJIS might be detecting synovitis that is not evident through swollen joint count, the distributions of ThermoJIS were compared using the aggregate of both at baseline and week 12 visits in two groups of patients: those with SJC28 = 0 and PGA > 5, and those with SJC28 = 0 and PGA ≤ 5 (Figure 2). The results show that ThermoJIS values are significantly higher in patients with PGA > 5 compared with those with PGA ≤ 5. Furthermore, a Spearman’s correlation coefficient of 0.30 (*p* = 0.03) is observed between ThermoJIS and PGA in patients with SJC28 ≤ 1.

### 3.4. Association of the Change of ThermoJIS with the Change of Swollen Joint Count

**Figure 3:**
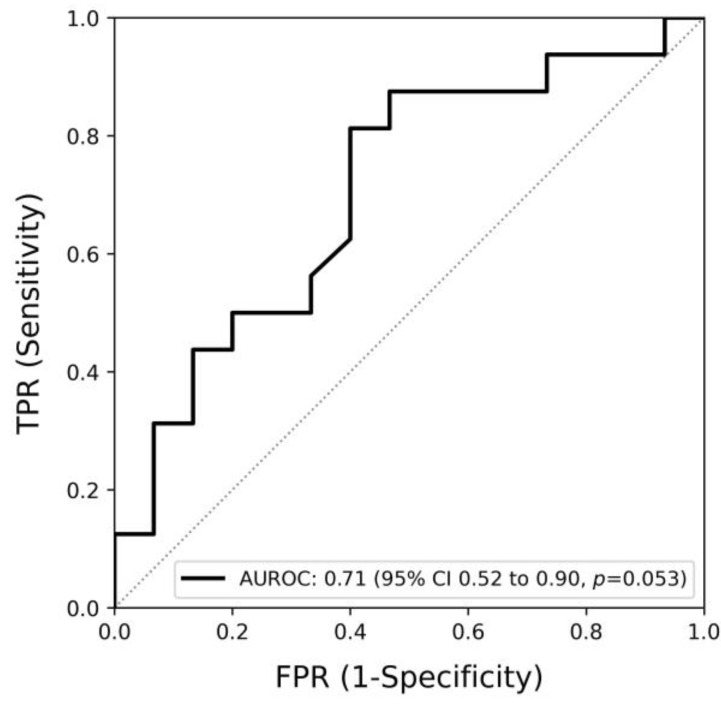
The area under the receiver operating curve (AUROC) of the Thermographic Joint Inflammation Score (ThermoJIS) for detecting transitions from 28-swollen joint counts (SJC28) > 1 at the baseline visit to SJC28 ≤ 1 at the week 12 visit (AUROC, 0.71; 95% CI 0.52–0.90, *p* = 0.053) (n = 31).

The Spearman’s correlation between the difference of ThermoJIS at baseline and the week 12 visit and the difference in SJC28 is 0.18 (*p* = 0.125), indicating a weak and non-significant correlation. However, the difference in ThermoJIS is found to have an AUROC of 0.71 (95% CI, 0.52 to 0.90, *p* = 0.053) for detecting when a patient transitions from SJC28 > 1 to SJC28 ≤ 1 (Figure 3). The number of patients with SJC28 > 1 at baseline was 31.

### 3.5. Correlation of ThermoDAI and ThermoDAI-CRP with CDAI, SDAI, and DAS28-CRP

**Figure 4:**
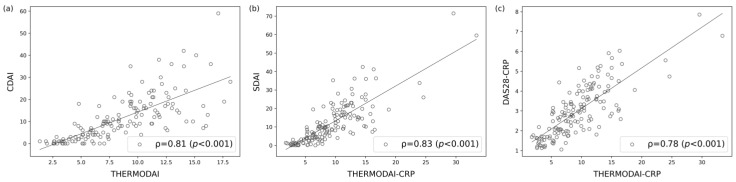
Correlation between the Thermographic Disease Activity Index (ThermoDAI) and Thermographic Disease Activity Index-C-reactive protein (ThermoDAI-CRP) with common indices used in clinical practice. (**a**) Spearman’s correlation between the ThermoDAI and Clinical Disease Activity Index (CDAI) (ρ = 0.81; *p* < 0.001); (**b**) Spearman’s correlation between the ThermoDAI-CRP and Simplified Disease Activity Index (SDAI) (ρ = 0.83; *p* < 0.001); (**c**) Spearman’s correlation between the ThermoDAI-CRP and 28-joint Disease Activity Score C-reactive protein (DAS28-CRP) (ρ = 0.78; *p* < 0.001). Aggregated data from baseline visit and week 12 visit (n = 150).

ThermoDAI and ThermoDAI-CRP show a strong Spearman’s correlation with CDAI (ρ = 0.81; *p* < 0.001) and SDAI (ρ = 0.83, *p* < 0.001), respectively (Figure 4a,b). ThermoDAI-CRP also show a strong correlation with DAS28-CRP (ρ = 0.78, *p* < 0.001) (Figure 4c). The current results are in line with prior studies regarding ThermoDAI and ThermoDAI-CRP [24].

### 3.6. Correlations of the Change of ThermoDAI and ThermoDAI-CRP with the Change of CDAI, SDAI, and DAS28-CRP

**Figure 5:**
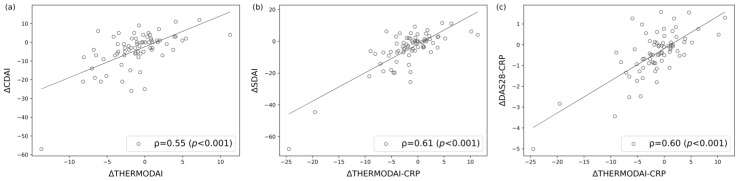
Correlation of the change in Thermographic Disease Activity Index (ThermoDAI) and Thermographic Disease Activity Index-C-reactive protein (ThermoDAI-CRP) between the baseline visit and the week 12 visit with the Clinical Disease Activity Index (CDAI), Simplified Disease Activity Index (SDAI), and 28-joint Disease Activity Score C-reactive protein (DAS28-CRP) (n = 73). (**a**) Spearman’s correlation between the ΔThermoDAI and ΔCDAI (ρ = 0.55, *p* < 0.001); (**b**) Spearman’s correlation between the ΔThermoDAI-CRP and ΔSDAI (ρ = 0.61, *p* < 0.001); (**c**) Spearman’s correlation between ΔThermoDAI-CRP and ΔDAS28-CRP (ρ = 0.60, *p* < 0.001).

The change in ThermoDAI and ThermoDAI-CRP from the baseline visit to the week 12 visit shows a moderate to strong Spearman’s correlation with the corresponding change in CDAI (ρ = 0.55, *p* < 0.001), SDAI (ρ = 0.61, *p* < 0.001), and DAS28-CRP (ρ = 0.60, *p* < 0.001) (Figure 5).

### 3.7. Contribution of ThermoJIS Component in ThermoDAI and ThermoDAI-CRP Indices

**Figure 6:**
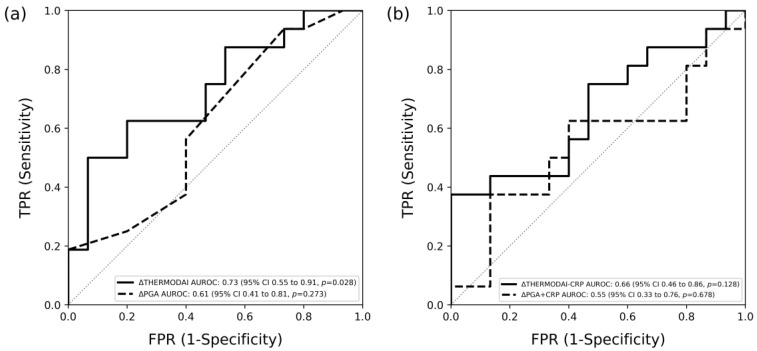
The area under the receiver operating curve (AUROC) of the Thermographic Disease Activity Index (ThermoDAI), Patient Global Assessment (PGA), and C-reactive protein (CRP) for detecting transitions from 28-swollen joint counts (SJC28) > 1 at the baseline visit to SJC28 ≤ 1 at the week 12 visit (n = 31). (**a**) AUROC of the ΔThermoDAI [0.73 (95% CI, 0.55 to 0.91, *p* = 0.028)] and ΔPGA [0.61 (95% CI, 0.41 to 0.81, *p* = 0.273)]; (**b**) AUROC of the ΔThermoDAI-CRP [0.66 (95% CI, 0.46 to 0.86, *p* = 0.128)] and ΔPGA + CRP [0.55 (95% CI, 0.33 to 0.76, *p* = 0.678)].

The changes in ThermoDAI and ThermoDAI-CRP were positively correlated with the change in SJC28 between the baseline and week 12 visit, with Spearman’s correlation coefficients of 0.32 (*p* = 0.006) and 0.35 (*p* = 0.003), respectively. In contrast, the correlations between the changes in PGA and PGA + CRP and the change in SJC28 are weaker, with correlation coefficients of 0.28 (*p* = 0.017) and 0.16 (*p* = 0.175), respectively. Furthermore, we assessed the AUROC of ThermoDAI, PGA, ThermoDAI-CRP, and PGA + CRP in detecting when a patient transitions from SJC28 > 1 to SJC28 ≤ 1. ThermoDAI has an AUROC of 0.73 (95% CI, 0.55 to 0.91, *p* = 0.028), while PGA has an AUROC of 0.61 (95% CI, 0.41 to 0.81, *p* = 0.273). ThermoDAI-CRP has an AUROC of 0.66 (95% CI, 0.46 to 0.86, *p* = 0.128), and PGA + CRP has the lowest AUROC of 0.55 (95% CI, 0.33 to 0.76, *p* = 0.678) (Figure 6).

### 3.8. Detection of Patients Who Met Treatment Response Using ThermoDAI and ThermoDAI-CRP

**Figure 7:**
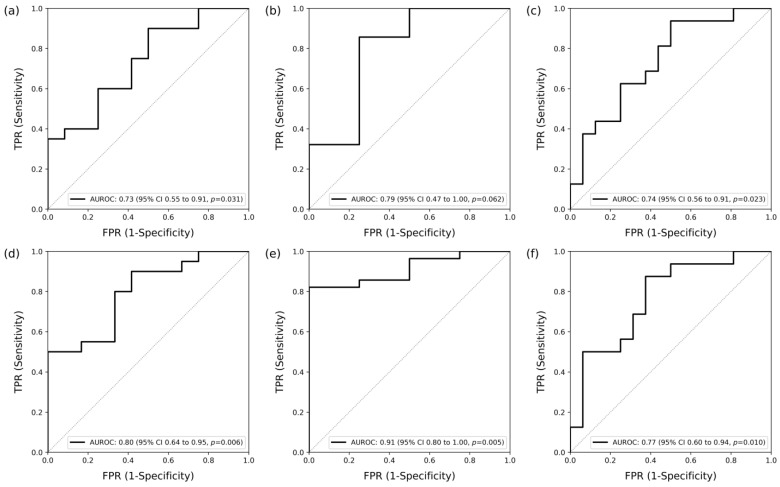
Detection of patients who met the American College of Rheumatology 20% response (ACR20), the American College of Rheumatology 50% response (ACR50), and European Alliance of Associations for Rheumatology (EULAR) treatment response criteria using the Thermographic Disease Activity Index (ThermoDAI) and Thermographic Disease Activity Index-C-reactive protein (ThermoDAI-CRP) (n = 32). (**a**) Area under the receiver operating curve (AUROC) of the ThermoDAI for detecting patients who met ACR20 [0.73 (95% CI, 0.55 to 0.91, *p* = 0.031)]; (**b**) area under the receiver operating curve (AUROC) of the ThermoDAI for detecting patients who met ACR50 [0.79 (95% CI, 0.47 to 1.00, *p* = 0.062)]; (**c**) area under the receiver operating curve (AUROC) of the ThermoDAI for detecting patients who met EULAR treatment response [0.74 (95% CI, 0.56 to 0.91, *p* = 0.023)]; (**d**) area under the receiver operating curve (AUROC) of the ThermoDAI-CRP for detecting patients who met ACR20 [0.80 (95% CI, 0.64 to 0.95, *p* = 0.006)]; (**e**) area under the receiver operating curve (AUROC) of the ThermoDAI-CRP for detecting patients who met ACR50 [0.91 (95% CI, 0.80 to 1.00, *p* = 0.005)]; (**f**) area under the receiver operating curve (AUROC) of the ThermoDAI-CRP for detecting patients who met EULAR treatment response [0.77 (95% CI, 0.60 to 0.94, *p* = 0.010)].

Treatment response analysis was performed in patients with moderate or high SDAI activity (SDAI > 11). The number of patients meeting this criterion is 32. ThermoDAI has an AUROC of 0.73 (95% CI, 0.55 to 0.91, *p* = 0.031) for ACR20 treatment response (Figure 7a), 0.79 (95% CI, 0.47 to 1.00, *p* = 0.062) for ACR50 treatment response (Figure 7b), and 0.74 (95% CI, 0.56 to 0.91, *p* = 0.023) for EULAR treatment response (Figure 7c). ThermoDAI-CRP has an AUROC of 0.80 (95% CI, 0.64 to 0.95, *p* = 0.006) for ACR20 treatment response (Figure 7d), 0.91 (95% CI, 0.80 to 1.00, *p* = 0.005) for ACR50 treatment response (Figure 7e), and 0.77 (95% CI, 0.60 to 0.94, *p* = 0.010) for EULAR treatment response (Figure 7f).

## 4. Discussion

External validation is an essential step in the development of any machine learning model before it is clinically applied, as it ensures that study findings are reproducible across different populations. In this study, we evaluated the performance of ThermoJIS, ThermoDAI, and ThermoDAI-CRP [23,24] in a new longitudinal cohort of RA patients from three hospitals.

ThermoJIS was originally trained to detect active synovitis in the hands using ultrasound as the gold standard technique [23]. Because this study used SJC28 instead of ultrasound as the criterion for joint inflammation, which is less sensitive and accurate, lower performance metrics than previous ultrasound-based studies were expected [30,31,32,33,34,35]. Additionally, SJC28 includes joints outside the hands that are not evaluated by ThermoJIS, such as shoulders, elbows, and knees [36]. Despite this, the results show the ability of ThermoJIS to detect joint inflammation. Furthermore, even though the correlation between changes in ThermoJIS and changes in SJC28 was weak, ThermoJIS was able to detect patients with swollen joints at the first visit who became inflammation-free at the later visit. These findings suggest the robustness of ThermoJIS and provide evidence of its validity. However, further research is needed to fully evaluate its performance using ultrasound as a criterion of validity.

In our previous study, ThermoJIS was significantly higher if active synovitis was detected using ultrasonography in clinical remission patients, regardless of the definition used, showing that ThermoJIS was sensitive enough for detecting subclinical synovitis [23]. In the present study, patients without swollen joints might still have joint inflammation. To examine this, we compared patients with SJC28 of 0 and PGA of 5 or less with patients with SJC28 of 0 and PGA greater than 5, hypothesising that PGA may indicate underlying inflammation. As expected, in the group of patients with SJC28 of 0 and PGA greater than 5, ThermoJIS levels were significantly higher. These findings suggest that, in certain cases, the false positives may actually be true positives, and that could be a cause of reduced performance metrics. Moreover, these results suggest that in some cases, ThermoJIS was detecting inflammation that the rheumatologist was not able to detect in the physical examination (subclinical synovitis), consolidating the evidence that this new technique could be useful in routine clinical practice to help the rheumatologist in the detection of joint inflammation. Subclinical synovitis has been associated with a higher risk of flares, progression of structural damage, and unsuccessful drug tapering, especially when Doppler activity is present [37,38]. Ultrasonography and Magnetic Resonance Imaging are sensitive methods for evaluating clinical and subclinical synovitis in RA, although routine use of these techniques is not feasible for most outpatient visits [39,40]. Evaluating synovitis with ThermoJIS is non-invasive, instantaneous, and automatic, making this tool accessible and suitable for routine use in clinical practice.

The thermographic disease activity indices, ThermoDAI and ThermoDAI-CRP, showed strong and consistent correlations with commonly used disease activity indices. Notably, the correlations between changes (from baseline to the week 12 visit) in ThermoDAI and ThermoDAI-CRP versus changes in SJC28 were found to be stronger than those between PGA and PGA + CRP versus SJC28, indicating their utility to detect changes in joint inflammation and disease activity over time when physical examination is not possible, even at the patient’s home, enabling remote assessment of RA patients. Furthermore, these indices were able to detect patients who met ACR20, ACR50, and EULAR treatment response criteria. This result suggests that ThermoDAI and ThermoDAI-CRP are promising new indices suitable for continuously monitoring patients in remote clinical trials, providing more information about their response to new treatments.

This study is subject to limitations. Although we have externally validated the pre-trained machine learning model in a longitudinal cohort, a larger cohort using hand ultrasound as a reference would elucidate the actual performance of the technique, particularly considering the limited sensitivity of physical examination and the fact that the 28 joints examined include some that are outside the hands and cannot be evaluated with thermography. Additionally, the imbalance in the recruited population introduces a potential limitation, as only 35% of patients in our cohort exhibited inflammation compared with 53% in the previous study. This difference may be partially attributed to the inclusion of subclinical cases. Furthermore, in this study, the average changes in TJC28, SJC28, and ThermoJIS between the baseline visit and the 12-week visit were relatively small, whereas there was a significant decrease in disease activity. Thus, it is likely that correlations between the change in ThermoDAI and ThermoDAI-CRP with the change in CDAI and SDAI were mainly driven by the change in PGA and CRP levels. To address this, future studies should aim to include a more balanced cohort of patients with a higher change in TJC28 and SJC28 between visits. Therefore, future research and development should include larger and more diverse cohorts of patients to ensure the generalisability and reliability of the performance outcomes. In addition, cost-effectiveness and impact studies should be conducted to evaluate the integration of thermography into routine clinical workflows.

## 5. Conclusions

The results of this external validation support the effectiveness of the Thermographic Joint Inflammation Score (ThermoJIS) in detecting joint inflammation in RA patients. In addition, both the Thermographic Disease Activity Index (ThermoDAI) and Thermographic Disease Activity Index C-reactive protein (ThermoDAI-CRP) have shown construct validity and sensitivity to change, indicating their usefulness in assessing the disease activity and treatment response in patients with RA.

Thermography is a rapid, portable, non-invasive, and affordable imaging technique that provides significant advantages for routine use. Moreover, the integration of machine learning allows for automatic and objective results without requiring extensive training, improving accessibility. These results provide encouraging evidence for the use of these novel indices as a complementary tool in routine clinical practice and in clinical trials for the evaluation of RA patients.

## Figures and Tables

**Table 1:** Patient characteristics.

	Baseline Visit (n = 77)	Week 12 Visit (n = 73)	*p*-Values
Age (years)	56 ± 13	56 ± 13	1.0
Female	60 (77.9%)	58 (79.5%)	1.0
Prednisone ≥ 7.5 mg	10 (13%)	6 (8.2%)	0.21
cDMARD	50 (64.9%)	52 (71.2%)	0.16
bDMARD/tsDMARD	35 (45.5%)	33 (45.2%)	0.32
TJC28	1 (0, 4)	1 (0, 3)	<0.05
SJC28	1 (0, 3)	0 (0, 2)	<0.05
SJC28 > 1	31 (40.3%)	22 (30.1%)	0.05
PGA	5 (2, 6)	3 (1, 5)	<0.05
EGA	3 (1, 5)	2 (0, 5)	<0.05
HAQ-DI	0.2 (0.0, 1.0)	0.1 (0.0, 0.6)	<0.05
ESR (mm/h)	19 (10, 36)	18 (10, 32)	0.08
CRP (mg/L)	3.8 (1.2, 8.0)	2.8 (1.0, 6.7)	0.52
DAS28	3.7 ± 1.4	3.3 ± 1.1	<0.05
CDAI	13.0 ± 11.9	9.2 ± 8.0	<0.05
SDAI	14.2 ± 13.4	9.9 ± 8.3	<0.05
ThermoJIS	4.7 ± 2.1	4.4 ± 1.7	0.35
ThermoDAI	8.9 ± 4.0	7.9 ± 3.6	<0.05
ThermoDAI-CRP	10.1 ± 5.5	8.5 ± 4.1	<0.05

Distributions are presented as mean ± SD or median (IQR). cDMARD, conventional DMARD (methotrexate, leflunomide, hydroxychloroquine, and sulfasalazine); bDMARD, biologic DMARD (abatacept, tocilizumab, etanercept, adalimumab, infliximab, certolizumab, golimumab, sarilumab, anakinra, apremilast, Ixekizumab, Secukinumab, Ustekinumab, and rituximab); tsDMARD, targeted synthetic DMARD (tofacitinib, baricitinib, and upadacitinib); TJC, tender joint count; SJC, swollen joint count; PGA, Patient Global Assessment; EGA, Evaluator Global Assessment; HAQ-DI, Health Assessment Questionnaire Disability Index; ESR, erythrocyte sedimentation rate; CRP, C-reactive protein; DAS28, 28-joint Disease Activity Score; CDAI, Clinical Disease Activity Index; SDAI, Simplified Disease Activity Index; ThermoJIS, Thermographic Joint Inflammation Score; ThermoDAI, Thermographic Disease Activity Index; and ThermoDAI-CRP, Thermographic Disease Activity Index-C-reactive protein. *p*-values calculated with the paired data (n = 73).

## Data Availability

The machine learning model and datasets presented are not readily available due to privacy restrictions.

## Supplementary Materials

The following supporting information can be downloaded at: https://www.mdpi.com/article/10.3390/diagnostics14131394/s1, Figure S1: Image of the thermal camera and the mobile application; Figure S2: Distribution of ThermoJIS, ThermoDAI, and ThermoDAI-CRP values at baseline visit and week 12 visit. Table S1: Individual patient clinical assessment, laboratory results, and ThermoJIS, ThermoDAI, and ThermoDAI-CRP values at baseline and week 12 visit.

## Appendix A. Previous Development of the Machine Learning Model

Thermal images of the development set [23] were resized to 160 × 120 pixels and processed to set the intensity values to an eight-bit grey-scale image. Additionally, images were improved by means of noise reduction, background removal, and contrast enhancement. For each thermal image, a set of regions of interest (ROIs) was obtained. ROIs were defined as local regions of the image with a large variation in intensity in all directions, such as corners or blobs. The corners or blobs were detected in various sizes using a scale-space representation of the image. Local thermal features were extracted using scale- and rotation-invariant descriptors from the ROIs detected. These features contained highly distinctive patterns, which may elucidate the underlying inflammatory process.

Each thermal feature obtained from the development set was labelled with the power Doppler (PD) sum score of the hand of the patient from whom the feature was extracted. A k-nearest neighbours algorithm (machine learning) was used to evaluate whether a new feature outside the development set was characteristic of synovitis (i.e., the average score of the k-nearest features of the development set was used to assign a synovitis score to new features). A Thermographic Joint Inflammation Score (ThermoJIS) was assigned to patients of the validation set by averaging the synovitis scores of its features. The hyperparameters (e.g., number of neighbours) were tuned using leave-one-out cross-validation within the development set. The ThermoJIS raw values of the validation set were normalised to be in a more intuitive range (mean of 5 and a standard deviation of 2.5).
