# Supplementary material for: External Validation of the Machine Learning-Based Thermographic Indices for Rheumatoid Arthritis: A Prospective Longitudinal Study
Source: Diagnostics (Basel). 2024 Jun 30;14(13):1394. doi: 10.3390/diagnostics14131394 (PMC11241557; doi:10.3390/diagnostics14131394)
Supplement: Supplementary file 1 [file diagnostics-14-01394-s001.zip › diagnostics-3057869-supplementary.pdf]

External Validation of the Machine Learning-Based Thermographic Indices for Rheumatoid Arthritis: A Prospective Longitudinal Study

Isabel Morales-Ivorra [1*], Delia Taverner [2], Oriol Codina [3], Sonia Castell [3], Peter Fischer [4], Derek Onken [5], Píndaro Martínez-Osuna [4], Chakib Battioui [5] and Manuel Alejandro Marín-López [6]

Supplementary Materials

Supplementary Figure S1: Image of the thermal camera and the mobile application that was developed to perform hand thermography.

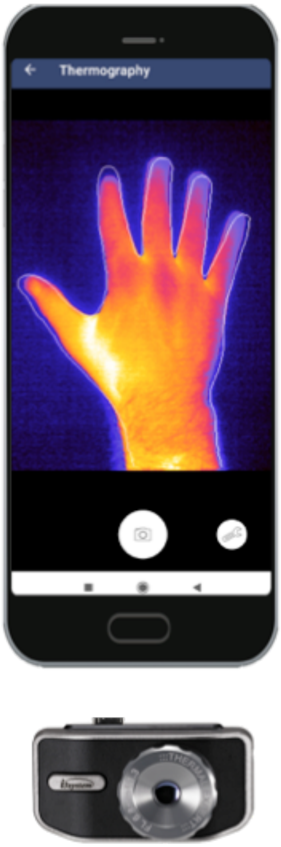

Supplementary Figure S2: Distribution of ThermoJIS, ThermoDAI and ThermoDAI-CRP values at baseline visit and week 12 visit.

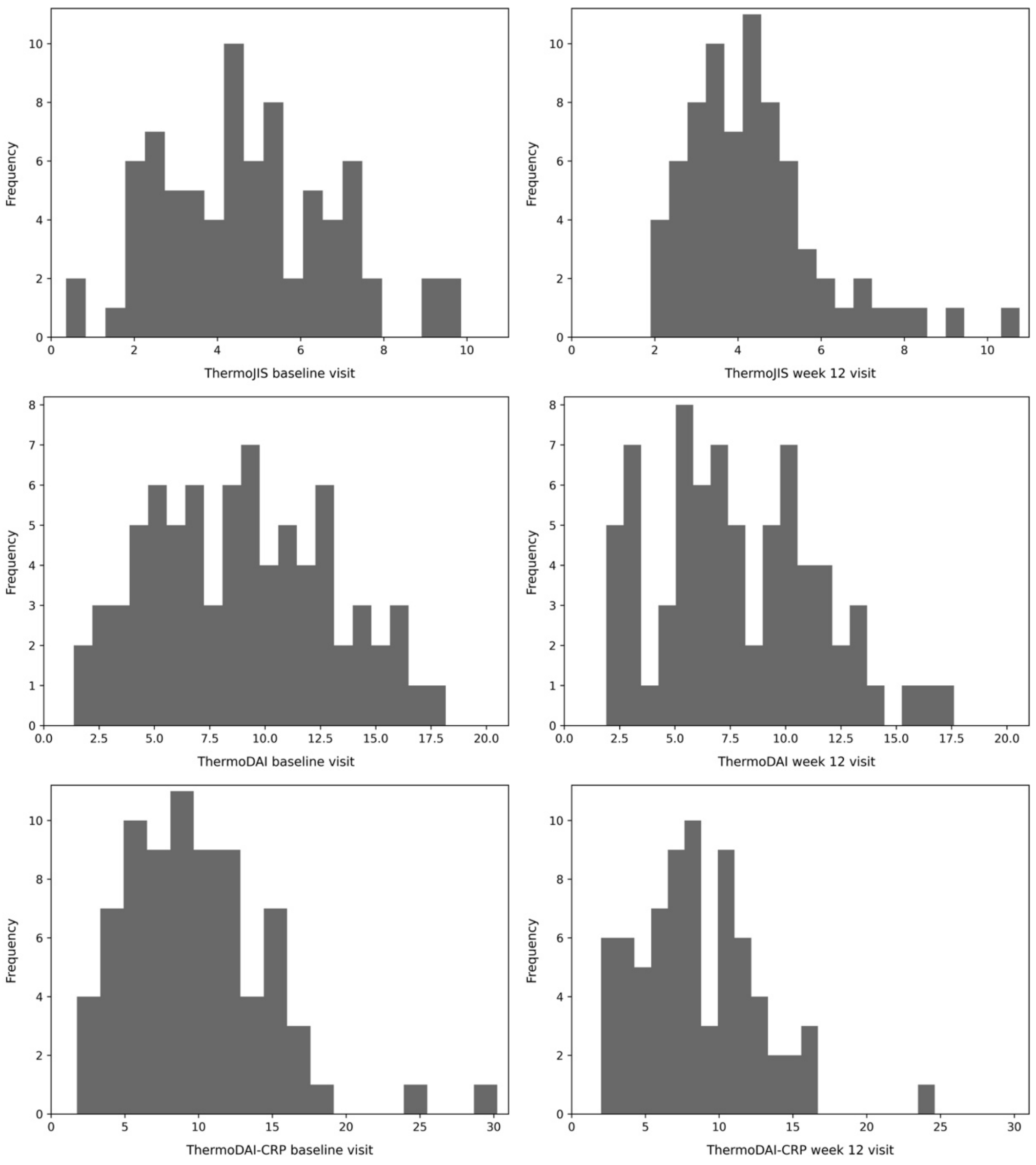

Supplementary Table S1: Individual patient clinical assessment, laboratory results, and ThermoJIS, ThermoDAI, and ThermoDAI-CRP values at baseline and week 12 visit.

	Baseline Visit										Week 12 Visit									
Patient	TJC28	SJC28	PGA	EGA	HAQ	CRP	ESR	ThermoJIS	ThermoDAI	ThermoDAI-CRP	TJC28	SJC28	PGA	EGA	HAQ	CRP	ESR	ThermoJIS	ThermoDAI	ThermoDAI-CRP
1	21	18	10	10	2.25	125	88	7.09	17.09	29.59	1	1	0	0	0	17	40	3.42	3.42	5.12
2	4	7	9	8	2.63	58	19	9.16	18.16	23.96	6	4	7	7	3	36	15	4.41	11.41	15.01
3	6	4	7	7	2.8	7.8	107	7.23	14.23	15.01	3	3	6	6	1.625	31	68	7.08	13.08	16.18
4	0	0	5	1	0.1	3.34	33	7.60	12.60	12.93	3	1	4	4	0.5	5.56	23	2.43	6.43	6.99
5	1	1	5	3	0	1.74	10	9.86	14.86	15.04	3	2	5	5	0.875	2.50	10	8.46	13.46	13.71
6	0	0	5	2	1.5	0.5	17	7.44	12.44	12.49	3	0	5	2	0.875	0.5	10	3.31	8.31	8.36
7	0	0	6	3	2	12	43	4.17	10.17	11.37	4	2	6	6	1.875	14	14	3.40	9.40	10.80
8	1	1	4	2	0	1.77	6	4.45	8.45	8.62	0	1	0	0	0	1.90	5	4.50	4.50	4.69
9	4	2	5	5	0.625	1.89	18	5.72	10.72	10.91	3	1	3	3	0.375	1.03	9	4.09	7.09	7.20
10	6	3	3	5	0.875	61	34	3.59	6.59	12.69	0	0	1	0	0	23	26	4.98	5.98	8.28
11	15	10	5	5	0.25	2.42	8	4.36	9.36	9.60	3	1	3	2	0	6.39	9	4.58	7.58	8.21
12	14	8	9	9	2.379	12	11	6.14	15.14	16.34	8	3	4	4	0.625	24	23	5.32	9.32	11.72
13	0	0	0	0	0.25	1.44	8	2.89	2.89	3.04	0	0	0	0	0.5	2.04	9	2.63	2.63	2.83
14	2	2	6	4	1	0.5	18	7.64	13.64	13.69	5	2	6	6	0.875	0.93	18	4.19	10.19	10.28
15	0	0	7	2	0.25	0.5	19	4.26	11.26	11.31	0	0	1	0	0	0.5	16	2.19	3.19	3.24
16	0	0	3	3	0.5	5.96	44	2.85	5.85	6.45	0	0	0	0	0.125	6.18	34	5.28	5.28	5.90
17	6	4	4	5	0.125	8.68	27	4.65	8.65	9.52	4	3	2	2	0	0.5	6	5.80	7.80	7.85
18	11	8	9	8	1.125	1.98	7	3.97	12.97	13.17	4	1	5	5	0.375	18	23	4.99	9.99	11.79
19	0	0	1	1	0.125	7.63	42	4.40	5.40	6.16	1	1	3	2	0	2.20	25	2.88	5.88	6.10
20	0	5	0	0	0	11	28	6.33	6.33	7.43	0	1	0	2	0	26	21	5.62	5.62	8.22
21	1	1	1	1	0.125	0.5	9	5.06	6.06	6.11	0	0	1	1	0	0.5	18	2.92	3.92	3.97
22	15	10	9	8	2.375	4.82	20	5.04	14.04	14.52	10	4	7	6	1.75	7.06	17	5.12	12.12	12.83
23	1	0	6	2	0.375	0.5	9	5.20	11.20	11.25	2	0	1	1	0.125	0.5	7	9.29	10.29	10.34
24	3	6	7	7	0.75	0.5	13	5.52	12.52	12.57	4	2	5	5	0.375	0.5	15	4.81	9.81	9.86
25	1	1	5	3	0.625	1.19	21	2.21	7.21	7.32	4	2	8	7	1.5	6.74	23	3.28	11.28	11.96
26	13	4	6	5	2	0.52	4	4.38	10.38	10.43	7	2	7	5	0.75	2.08	7	5.71	12.71	12.92
27	6	6	3	3	2.25	15	19	1.80	4.80	6.30	9	4	9	8	1.75	6.57	7	3.02	12.02	12.68
28	6	4	4	4	0.25	0.5	7	7.13	11.13	11.18	2	1	5	4	0	1.74	9	4.85	9.85	10.03
29	0	1	8	5	0.75	0.92	19	1.90	9.90	9.99	0	2	7	6	0.875	0.92	19	4.53	11.53	11.62
30	0	9	4	4	0.75	3.67	112	5.35	9.35	9.72	2	10	7	5	0.25	3.67	112	4.21	11.21	11.58
31	6	5	7	7	0.375	11	65	6.29	13.29	14.39	0	0	2	2	0.25	0.5	20	3.13	5.13	5.18
32	7	4	5	5	0.625	0.84	9	2.93	7.93	8.01	3	0	0	0	0.25	1.02	12	2.84	2.84	2.94
33	14	3	9	9	2.75	10	30	5.06	14.06	15.06	7	3	7	6	0.875	0.5	16	3.97	10.97	11.02
34	0	0	3	2	0	20	19	3.95	6.95	8.95	0	0	0	0	0	4	17	3.42	3.42	3.82
35	2	2	2	2	0	3.6	15	7.11	9.11	9.47	0	0	0	1	0.25	2.8	18	2.66	2.66	2.94
36	0	0	2	2	0	1.1	5	3.17	5.17	5.28	0	1	0	0	0	1.1	4	3.32	3.32	3.43
37	0	0	0	0	0	0.8	34	3.80	3.80	3.88	0	0	0	0	0	0.6	19	3.35	3.35	3.41
38	2	2	5	3	0.5	25.09	72	6.92	11.92	14.43	1	0	5	5	0.25	20.8	61	3.34	8.34	10.42
39	1	0	0	0	0.125	4	6	4.24	4.24	4.64	2	0	1	0	0	17.6	32	4.05	5.05	6.81
40	0	0	1	0	0.25	13.5	58	3.23	4.23	5.58										
41	0	0	0	0	0	1	41	3.60	3.60	3.70	0	0	0	0	0	0.6	33	2.57	2.57	2.63
42	0	0	2	1	0	2.5	10	2.07	4.07	4.32	0	0	3	1	0.125	5.6	30	3.05	6.05	6.61
43	0	2	1	1	0	3.8	34	5.21	6.21	6.59	0	2	3	3	0.375	12.5	63	3.89	6.89	8.14
44	3	2	2	2	0.25	9.3	36	5.31	7.31	8.24	1	1	2	2	0	2.7	30	4.03	6.03	6.30
45	4	1	6	2	0.375	0.5	10	2.31	8.31	8.36	3	2	5	2	0.25	1.2	20	2.31	7.31	7.43
46	2	2	5	5	0.75	7.4	81	6.72	11.72	12.46	5	0	6	5	0.625	5	91	4.52	10.52	11.02
47	12	12	7	7	1.125	215.4	120	4.86	11.86	33.40	2	2	5	4	0.125	20.3	35	6.84	11.84	13.87
48	2	0	5	2	0	1.4	38	1.96	6.96	7.10	0	0	7	1	0	1.3	22	2.84	9.84	9.97
49	3	0	4	1	0	5.3	4	0.76	4.76	5.29	0	0	3	2	0	5.3	10	2.76	5.76	6.29
50	3	0	9	5	1.25	0.3	6	6.77	15.77	15.80	4	2	5	5	1.125	1.2	15	8.00	13.00	13.12
51	2	6	6	5	0.125	19.4	31	3.44	9.44	11.38	6	6	5	6	0.25	0.5	7	4.22	9.22	9.27
52	3	2	7	4	1.125	5.6	38	2.67	9.67	10.23	3	3	6	5	1.25	9.3	42	4.56	10.56	11.49
53	0	0	2	1	0.625	2.7	43	2.50	4.50	4.77	2	0	5	0	1.125	3.7	25	10.77	15.77	16.14
54	3	0	4	0	0.375	3.6	18	4.26	8.26	8.62	1	0	2	1	0	1.6	201	3.61	5.61	5.77
55	0	0	1	0	0	0.3	13	1.62	2.62	2.65	1	0	0	0	0	0.3	13	2.64	2.64	2.67
56	0	0	0	0	0	7	34	2.65	2.65	3.35	0	0	2	1	0	4.1	41	4.71	6.71	7.12
57	0	0	1	0	0	4	26	2.46	3.46	3.86	0	0	3	1	0	4	34	4.38	7.38	7.78
58	1	1	3	4	0	8	32	6.65	9.65	10.45										
59	0	0	0	0	0	10	2	6.11	6.11	7.11	0	0	0	0	0	0.29	2	6.64	6.64	6.67
60	6	0	8	3	1.6	0.39	3	4.41	12.41	12.45	1	0	10	2	0.250	0.57	2	6.16	16.16	16.21
61	1	2	2	4	0.25	8	15	2.43	4.43	5.23	1	2	2	1	0.125	1.2	5	3.14	5.14	5.26
62	11	0	6	4	0.125	7	10	5.37	11.37	12.07	0	0	1	1	0	7.8	13	3.76	4.76	5.54
63	19	3	7	7	2	3	16	9.43	16.43	16.73	9	1	5	7	0.250	2	7	4.43	9.43	9.63
64	3	1	7	7	125	18	74	5.65	12.65	14.45	1	1	10	7	1.625	70	52	7.61	17.61	24.61
65	0	0	0	0	0	3	44	5.12	5.12	5.42	0	0	0	0	0	2	63	5.15	5.15	5.35
66	4	0	4	2	0.125	4	12	4.32	8.32	8.72	2	0	5	3	0.125	7	36	4.31	9.31	10.01
67	0	0	1	0	0	4	8	0.36	1.36	1.76	0	0	1	0	0	3	6	2.06	3.06	3.36
68	0	0	5	0	1.125	1	2	3.94	8.94	9.04										
69	1	1	5	5	1.125	74	5	6.45	11.45	18.85										
70	0	0	0	0	0.125	8	18	7.04	7.04	7.84	0	0	2	1	0	0.49	8	5.35	7.35	7.39
71	0	0	7	1	0.625	5	31	9.01	16.01	16.51	0	0	8	2	1.125	5	29	6.28	14.28	14.78
72	0	0	0	0	0.125	5.57	163	1.99	1.99	2.54	0	0	2	0	0.125	3	19	5.43	7.43	7.73
73	0	0	5	3	0.25	7.9	36	3.59	8.59	9.38	0	0	3	1	0.125	4	17	3.49	6.49	6.89
74	3	1	5	4	0.125	0.96	24	5.34	10.34	10.43	1	1	3	3	0	1	12	4.68	7.68	7.78
75	0	0	5	5	0.250	1.51	9	2.37	7.37	7.52	1	0	0	0	0.125	1	12	1.90	1.90	2.00
76	4	0	2	1	0.125	3.79	52	2.97	4.97	5.35	1	0	1	0	0	17	55	3.79	4.79	6.49
77	3	2	2	3	0	3.7	24	5.00	7.00	7.37	2	0	3	1	0.125	5	37	4.91	7.91	8.41
